# Changes in Psychophysical Parameters in Seniors During the COVID-19 Pandemic—A Repeated Observational Study

**DOI:** 10.3390/healthcare14010084

**Published:** 2025-12-30

**Authors:** Dorota Szydłak, Ewelina Grabska-Klein, Anna Brzęk

**Affiliations:** Department of Physiotherapy, Faculty of Health Sciences in Katowice, Medical University of Silesia in Katowice, 40-752 Katowice, Poland; egrabska@sum.edu.pl (E.G.-K.); abrzek@sum.edu.pl (A.B.)

**Keywords:** seniors, COVID-19, physical activity, anxiety, depression, sleep disorders

## Abstract

Older individuals primarily belong to the group with an increased risk of SARS-CoV-2 infection. Social distancing and other strategies to mitigate the COVID-19 pandemic may negatively impact the psychophysical parameters of seniors. Objective: The main aim of this study is to analyze the changes in psychophysical parameters among seniors in the context of the COVID-19 pandemic over a one-year observation. Materials and methods: The observation involved 54 respondents aged 60 and above. The study was conducted three times from March 2020 to April 2021. The selected research method was a diagnostic survey based on a questionnaire. Results: The level of general physical activity in the study groups during the pandemic was determined to be moderate with an upward trend. A downward trend in self-assessment of quality of life was observed, particularly in the area of mental health, along with an increase in symptoms of anxiety, aggression, and insomnia. Conclusions: The pandemic period did not significantly affect the level of physical activity among the surveyed seniors, but an intensification of mental symptoms was noticeable.

## 1. Introduction

According to the World Health Organization (WHO), there is a close relationship between the intensity of daily physical activity and the overall psychophysical well-being among older individuals [1,2,3,4,5]. The current recommendations provided by the WHO regarding physical activity among seniors emphasize the importance of any physical activity undertaken. The report suggests that the level of weekly physical activity should involve moderate-intensity aerobic activity for 150–300 min or vigorous-intensity activity within the range of 75–150 min. Additionally, the report highlights the necessity of incorporating strength training involving major muscle groups for at least two days a week. Furthermore, the WHO recommendations indicate the possibility of implementing combined training, involving a mix of different activities in terms of their intensity and type [6]. Such an approach seems appropriate in ensuring an adequate level of physical and mental health, leading to a reduction in functional and cognitive impairments, falls and fracture risks, depression, geriatric syndromes, hospitalization frequency, and ultimately decreasing mortality among older individuals [1,7]. There are various predictors affecting the limitation of physical activity, including fear of movement, health status, the number of chronic diseases, or lack of a companion for exercise [8,9]. In 2020, an additional factor emerged in the form of the COVID-19 pandemic affecting the daily rhythm of behaviors, especially among the senior population. The situation forced many governmental organizations in several countries to implement appropriate legal regulations temporarily restricting the functioning of schools, workplaces, and sectors, like sports and recreation, commerce, gastronomy, and tourism. Additionally, many social restrictions or obligations were imposed, such as social distancing, mask-wearing, or isolation. These enforced behaviors undoubtedly had a significant impact on limiting the spread of the SARS-CoV-2 virus while simultaneously minimizing physical and mental functioning among the senior population, making them more susceptible to serious illnesses due to their age-related health condition. To prevent getting infected, many older individuals reduced their social interactions, affecting their regular activities and decreasing overall quality of life [10,11,12]. Moreover, due to measures like quarantines and social distancing, older people experienced increased feelings of loneliness and detachment or isolation, leading to loneliness, sadness, and a sense of helplessness [13,14]. Many older individuals also faced challenges related to technology because they were not accustomed to using computers or smartphones, making it difficult for them to stay connected virtually, further isolating them [15]. Given the above, the authors attempted to assess psychophysical parameters and their changes during the COVID-19 pandemic. They also sought to find a correlation between the level of physical activity and the well-being of seniors.

Regular physical activity plays a key role in maintaining physical function, psychological well-being, and independence in older adults. According to the World Health Organization (WHO), older individuals should engage in at least 150–300 min of moderate-intensity or 75–150 min of vigorous-intensity aerobic physical activity weekly, supplemented with muscle-strengthening activities [1,2,3,4,5,6]. These recommendations are specifically tailored to seniors and account for age-related physiological changes and comorbidities.

Despite the well-documented benefits of physical activity, many older adults experience barriers that limit regular movement, including chronic diseases, fear of injury, limited social support, and environmental constraints [7,8,9]. The COVID-19 pandemic introduced additional challenges, particularly affecting seniors. Government-imposed restrictions, lockdowns, and social distancing measures substantially altered daily routines and limited opportunities for physical, social, and recreational activities [10,11,12,13]. Numerous international studies conducted during the pandemic have reported reduced physical activity, increased sedentary behavior, and worsening mental health among older adults [14,15,16,17,18,19,20]. However, most of these studies were cross-sectional, relied on single-time-point online surveys, and often excluded the oldest-old population. Longitudinal studies using repeated measurements across different pandemic stages remain scarce, particularly those simultaneously assessing physical activity, quality of life, mental health, and sleep parameters.

Therefore, the aim of this study was to assess changes in psychophysical parameters—physical activity, quality of life, anxiety, depression, aggression, and sleep disturbances—among seniors during three distinct stages of the COVID-19 pandemic and to examine associations between physical activity and selected mental health indicators.

## 2. Materials and Methods

This study was designed as a repeated observational study without randomization and without a control group. Data were collected at three measurement time points corresponding to different stages of the COVID-19 pandemic.

Stage 1 (Study I)—March 2020, at the onset of the COVID-19 pandemic, before the first lockdown in Poland.

Stage 2 (Study II)—Around October and November 2020, during the second lockdown in Poland, six months after the restrictions were imposed.

Stage 3 (Study III)—April 2021, during the third lockdown in Poland, one year after the onset of the pandemic.

### 2.1. Participants

In total, 53 participants aged 60 to 93 years old took part in the observation (mean age = 69.6 ± 7.9), including 32 women and 21 men. Participants were recruited using convenience sampling and volunteered to participate.

Participants aged ≥80 years constituted 9.4% of the sample, including 3 women and 2 men. Individuals who did not report any chronic diseases were predominantly aged 60–67 years. The wide age range reflects real-world senior populations but introduces heterogeneity in functional capacity and subjective perception of physical activity. Due to the limited sample size, subgroup analyses by narrower age categories were not performed.

The inclusion criteria were as follows: age ≥ 60 years, independence in activities of daily living, good general health according to the WHO definition, and informed consent.

The exclusion criteria were the following: diagnosed dementia, active cancer, chest pain, recent fractures, severe musculoskeletal disorders, or refusal to participate. The characteristics of the study group are presented in Table 1.

During the pandemic, 24.52% of respondents lived alone, 52.83% with a spouse, and 22.64% with family. In terms of activity, 31.25% of females and 33.33% of males were professionally active, and 12.5% of women and 4.76% of men were socially active.

The clinical characteristics show that both females and males most often mentioned diseases of the circulatory system (53.13% females, 57.14% males) and musculoskeletal system diseases (40.76%, 23.81%) as their main diseases. Of the participants, 18.75% of female respondents and 19.05% of male respondents did not indicate any diseases. No participant was hospitalized due to COVID-19.

### 2.2. Methods

The research method utilized a diagnostic survey conducted through a questionnaire consisting of several sections. The first part of the questionnaire contained information regarding socio-demographic data, enabling the characterization of the studied group. Additionally, the survey questionnaire included specific questions aimed at deepening the understanding of the parameters selected by the authors. Subsequently, the following questionnaires were included in the survey:

1. The Modified Baecke’s Questionnaire for Older Adults allows for the assessment of physical activity in three aspects: household chores, sports activities, and recreational activities [17,18];
2. The SF-12v2 Health Survey (Short Form 36 Health Status Questionnaire) is used to assess the quality of life in two categories: physical and mental [19];
3. The Modified Hospital Anxiety and Depression Scale (HADS-M) is utilized to assess depression, anxiety, as well as aggression [20];
4. The Athens Insomnia Scale (AIS) is a self-report tool consisting of 8 questions regarding sleep time, sleep quality, onset, waking up, and psycho-physical well-being the next day [21,22].

Data were collected using a diagnostic survey administered online, by telephone, or by personal contact, depending on epidemiological restrictions. The survey included sociodemographic data and the following standardized instruments:

- Modified Baecke Questionnaire for Older Adults—assessment of habitual physical activity. Minor linguistic and contextual adaptations were introduced to reflect pandemic conditions [17,18,19].
- SF-12v2 Health Survey—assessment of health-related quality of life [20].
- Modified Hospital Anxiety and Depression Scale (HADS-M)—assessment of anxiety, depression, and aggression [21,22].
- Athens Insomnia Scale (AIS)—assessment of sleep quality and insomnia symptoms [23,24].

All questionnaires were used in their original, previously validated versions. The term ‘modified’ refers to established adaptations available in the literature; no modifications were introduced by the authors.

The Health Physical Activity (HPA) index was calculated individually for each participant based on the Modified Baecke Questionnaire and analyzed at the group level.

### 2.3. Statistical Method

The obtained results underwent statistical analysis using the STATISTICA 12 software. Descriptive statistics were presented using mean, standard deviation, median, quartiles, and interquartile range. The normality of the distribution in the studied group was assessed using the Shapiro–Wilk test, with a significance level set at *p* < 0.05. Furthermore, to compare the results, the ANOVA test for repeated measures and post hoc tests were employed. To determine the relationships between the variables under investigation, the R-Spearman method was used.

## 3. Results

### 3.1. The Physical Activity of the Surveyed Individuals and Its Changes During the Pandemic

**Figure 1:**
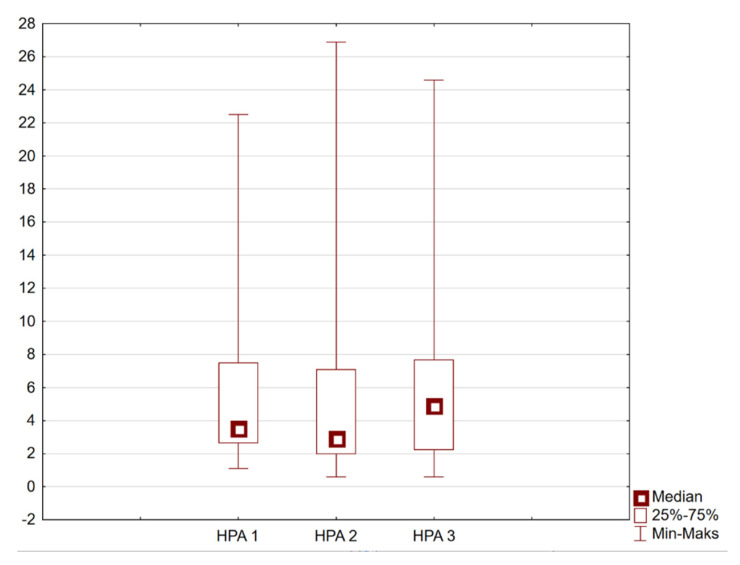
Mean Health Physical Activity (HPA) index for the entire study group at three measurement points: HPA1—Study I (March 2020); HPA2—Study II (October–November 2020); HPA3—Study III (April 2021). Individual HPA scores were averaged across all participants to present overall group-level trends.

Considering the entire group of participants, the overall level of physical activity improved (*p* = 0.0068, Figure 1), but no differences were noted in individual assessments and domains when divided by gender.

Physical activity levels remained above average across all three measurement points. Although a temporary decrease was observed during Study II, followed by an increase in Study III, these changes were not consistently statistically significant. Figure 1 presents the distribution of the HPA index at three time points (HPA1, HPA2, HPA3), corresponding to Studies I, II, and III, respectively.

Based on the overall HPA (Health Physical Activity) index, it can be inferred that the level of physical activity in the studied group was above average. The level of physical activity was indirectly determined by calculating quartiles, where Q1—low level of physical activity, Q2—average level of physical activity, and Q3—high level of physical activity. The HPA index was calculated for each individual and then averaged for each group, women and men (Table 2).

A decreasing trend in the overall HPA index is observed during the second lockdown in both genders. On the other hand, during the third lockdown, the HPA index was highest in both studied groups, indicating an increasing trend of the parameter over the course of the pandemic. However, these differences were not statistically significant, and the variability among individuals was considerable. Median and mean values suggest that physical activity levels may differ substantially depending on age, with younger seniors likely engaging in more intensive physical activity than the oldest participants. (c.f Table 2).

### 3.2. Analyzing the Quality of Life of the Respondents and Its Changes During the Pandemic

Analyzing the continuity of the obtained results, an overall decreasing trend was observed in almost all domains for both genders. The only exception was the physical health domain among men, which showed an increasing trend. Statistically significant differences occurred only in the female group for the MCS parameter (0.02). However, individual variability was considerable, and many observed changes were not statistically significant. The detailed analysis of the quality of life during the pandemic is presented in Table 3.

### 3.3. Anxiety, Depression, Aggression, and Insomnia Among the Surveyed Individuals and Their Changes During the Pandemic

From the analysis of the HADS questionnaire, it appeared that the pandemic intensified feelings of anxiety in both studied groups. Statistically significant differences between the studies were observed only in the female group (*p* = 0.004). It was also noticeable that women exhibited higher levels of anxiety than men (Table 4).

In both studied groups, a decrease in symptoms of depression was observed during the pandemic. Statistically significant differences between the studies were observed only in the male group (*p* = 0.02). Symptoms of depression were at a similar level in both studied groups (Table 4).

During the pandemic in both studied groups, an exacerbation of irritability symptoms was noticeable. Comparing the continuity of the study (Study I–Study III), there is an increasing trend in the parameter being studied in women, while in men, values returned to the initial level. However, statistically significant differences occurred only in the female group (*p* = 0.03). Female respondents also exhibited higher levels of irritability (Table 4).

The analysis of the AIS questionnaire indicated an intensification of symptoms of insomnia in both studied groups during the pandemic. However, a higher level of sleep problems was characteristic of female respondents (Table 4).

In summary, an increase in anxiety, irritability, and insomnia symptoms was observed during the pandemic, particularly during the second lockdown. In men, anxiety levels did not show a consistent increasing trend across all stages and should therefore be interpreted cautiously. Depression symptoms remained relatively stable or decreased slightly over time in both sexes.

### 3.4. Correlations Between Level of Physical Activity and Quality of Life, Anxiety, Depression, Aggression, or Sleep Disorders

In general, no statistically significant correlations were observed between the level of physical activity and quality of life, anxiety, depression, aggression, or sleep disorders in any of the surveyed groups across the three studies. This indicates that, for the majority of participants, physical activity did not demonstrate a measurable association with psychological or functional outcomes. Two exceptions were identified. In Study I, a significant correlation was detected between physical activity and depression in the group of women, suggesting that higher levels of physical activity were associated with lower depressive symptoms in this subgroup. Similarly, in Study III, a significant association between physical activity and depression was found among men. These findings imply that, although the general pattern across the studies did not support a relationship between physical activity and mental health indicators, depressive symptoms may be more sensitive to variability in physical activity levels in specific demographic groups (Table 5). The correlation analyses conducted in this study did not provide evidence of consistent associations between the level of physical activity and quality of life, anxiety, depression, aggression, or sleep disorders across the three measurement points in either women or men. Most correlation coefficients were small and did not reach statistical significance, indicating the absence of stable relationships at the group level. A small number of statistically significant correlations were observed; however, these occurred in different subgroups, were not reproduced across measurement points, and concerned only a single outcome variable. Given the limited sample size, the exploratory nature of the analyses, and the absence of correction for multiple testing, these isolated findings should be interpreted as descriptive and hypothesis-generating rather than confirmatory. Overall, the results of the present study do not allow for conclusions regarding a systematic relationship between physical activity level and psychophysical outcomes in older adults during the COVID-19 pandemic. Any potential associations require confirmation in future studies employing larger, well-defined cohorts and more rigorous analytical designs (Table 5).

## 4. Discussion

The coronavirus pandemic has forced many drastic changes in the daily functioning of the human population. Evidence of the impact of these changes on various aspects of human life and functioning in long-term observation is continually expanding with new knowledge [25,26]. Our own research includes observations of respondents during three periods of tightening restrictions during the pandemic and observation of these respondents in the post-pandemic period, which allows for taking appropriate steps to develop physioprophylaxis and education of seniors in the dissemination of knowledge and its practical application. As the pandemic situation has shown, it is extremely important to be able to benefit from acquired knowledge and previously established habits, especially in times of threat (pandemic).

It should be noted, however, that the study had an observational design, without a control group, randomization, or a clearly defined cohort strategy, which limits the ability to draw causal inferences. Additionally, methodological limitations, including the small sample size and the lack of correction for multiple comparisons, should be considered when interpreting the results.

Long-term pandemic restrictions were reflected in the performance of daily physical activity [27,28]. This was confirmed by the study by Kosowski et al., which indicated that the pandemic period interferes with the psycho-physical state of the respondents and affects their well-being [29]. Meanwhile, Ashdown-Franks et al. have proven that physical activity can serve as both a preventive measure and a supportive treatment for many mental disorders, which is particularly important during pandemic restrictions [30].

From our own research, it follows that the overall level of HPA in the studied group improved, but no differences were observed in individual assessments and domains divided by gender. Based on the overall HPA index, it can be inferred that the level of physical activity was above average and characterized by an upward trend. Based on our findings, the overall HPA index suggested an above-average level of physical activity in the study group, with a slight upward trend over time. However, no significant differences were observed by gender, and individual variability was considerable. The associations between physical activity and mental health outcomes were moderate and varied across demographic subgroups, suggesting that conclusions regarding increased physical activity should be drawn cautiously.

Szara et al. believe that pandemic-related restrictions affected the limitation of activity in cultural and sports centers, which directly engage older individuals in social, physical, and mental activities [31]. Research conducted by De Oliveira et al. indicated that restrictions on physical activity may predispose increased levels of anxiety and depression among seniors [32]. Similarly, Wasiuk et al. argued that a low level of physical activity predisposed the occurrence of many movement dysfunctions. Moreover, they showed that psychological factors, especially kinesiophobia, also contributed to physical inactivity, in addition to pain, fatigue, or disease factors [33]. Atici et al.’s research showed that during the pandemic, 15.6% of respondents attributed reduced physical activity to fear of falling and fear of movement itself [34]. Similarly, Hoffman’s research findings indicated that 36.9% of respondents reported a decrease in physical activity, while 35.1% reported an increase in sedentary behavior. Additionally, researchers noted that kinesiophobia during the pandemic was intensified by increased social isolation, worsened condition, and fear of falling [35]. Similar conclusions were drawn by Stanton et al., who noticed that part of the population exhibited autonomous regulation of physical activity to perform alternative activities at home. Additionally, it was observed that another part of the population developed new habits of physical activity because it was the only justified reason to leave home [36]. McCarthy et al. presented similar findings, demonstrating that individuals over the age of 65 remained active during the pandemic, and their activity increased as the restrictions eased [25].

Physical activity and self-assessment of health are directly related variables. Therefore, the emergence of the coronavirus pandemic also brought about changes in the aspect of quality of life assessment. According to Sacre et al., pandemic-related restrictions negatively impacted quality of life, certain behavioral risk factors, and access to healthcare [37]. Studies conducted by Briere et al. indicated that over 33% of respondents denied that the pandemic significantly affected their mental (35%) or physical (37.6%) health. However, the impact on quality of life was multifactorial, dependent on factors such as the number of coexisting diseases, loneliness, and financial limitations [38]. Nightingale et al. emphasized the importance and necessity of safely promoting physical activity during leisure time during the COVID-19 pandemic in the elderly population to support health-related quality of life [39].

The analysis of our own research revealed changes in the aspect of health-related quality of life among respondents over the course of a year. In the female group, a decrease in quality of life was observed in both domains, while in the male group, an increase in the physical domain and a decrease in the psychological domain were noted. Analysis of our data indicated that over the course of the year, there were changes in quality of life: in the female group, a decrease was observed in both domains, whereas in men, an increase was seen in the physical domain and a decrease in the psychological domain. It should be noted that most changes were not statistically significant, and individual differences were considerable.

Self-rated health is a variable influenced by many factors, both individual and environmental [40]. Research by Knapik et al. pointed to the significant impact of education level on self-rated health. Among those with lower general health scores, a lower level of education was observed [41]. However, Pacian et al. did not observe significant relationships between education and individual domains of quality of life [42]. These differences may stem from variations in the number of respondents as well as the use of different tools. This aspect was not analyzed in our own research.

The emotional state of the participants is influenced by numerous factors such as somatic diseases and age. In the study by Pacian et al., an increase in the occurrence of depression associated with age was observed. Additionally, depression was more common among respondents with higher education and professional status [42]. In the era of the pandemic, significant factors also seem to have been the limitations of the needs of elderly individuals in the psycho-social sphere in order to avoid illness. Ahmed et al. noticed that the appearance of the coronavirus did not significantly affect the exacerbation of depression and anxiety symptoms. The authors pointed out that in geriatric patients, the exacerbation of depression symptoms was associated with the occurrence of a stroke rather than pandemic-related factors. According to their research, the exacerbation of depression was associated with a lack of access to rehabilitation [43]. On the other hand, Chiaravalloti et al., analyzing the impact of the COVID-19 pandemic on patients with multiple sclerosis, did not find significant relationships between disease duration, processing speed ability, or EDSS, and changes in depression or anxiety symptoms. However, most patients reported the virus’s impact on their mental well-being [44]. The results of a cross-sectional study conducted by Ozdin et al. suggested that the groups most affected psychologically by the COVID-19 pandemic were women, individuals with pre-existing mental illnesses, people living in urban areas, and those with accompanying chronic illness [45].

Our own research indicated that the pandemic intensified feelings of anxiety, irritability, and sleep disturbances during the second lockdown, both in women and men, while these symptoms slightly decreased during the third lockdown. However, the observation of the continuity of the study showed an increasing trend in the observed variables, especially in the female group. The opposite situation was observed regarding depression. In the male group, a decrease in depression symptoms was observed during both the second and third lockdowns. On the other hand, in the female group, the level of depression initially decreased, but during the third lockdown, symptoms worsened. However, considering the continuity of the study, a decreasing trend of the discussed parameter could be observed in both examined groups. In both examined groups, depression symptoms were at a similar level. Additionally, in our own research, a correlation between HPA and depression was observed in the examined groups. In our data, anxiety, irritability, and sleep disturbances were elevated during the second lockdown in both women and men, followed by a slight decrease during the third lockdown. The depression trend was mixed: men showed a decrease in symptoms over time, while women initially improved, then slightly worsened in the third lockdown. Associations between physical activity and depression were present in selected demographic groups, but overall correlations were moderate and variable.

From the analysis of the available literature, it emerged that regardless of additional socio-demographic (age, gender, place of residence) or situational (pandemic) characteristics, depression was significantly correlated with physical activity [39,46]. The study by Pacian et al. indicated that respondents with higher scores in the psychological domain than in the physical domain were less at risk of depression [42]. Similar results were obtained by Patten et al., who demonstrated the positive impact of physical activity on mood improvement and thus the reduction in depression symptoms [47]. Nightingale et al. also observed that lower physical activity during the pandemic was significantly associated with the exacerbation of depression symptoms [39].

In the study by Pacian et al., no differences were observed between the risk of depression and the overall quality of life [42].

Currently, insomnia is also a serious health problem associated with a significant psychological burden. Voitsidis et al. believe that the main predictors of insomnia were pandemic-related anxiety, loneliness, and more severe depression symptoms [27]. They also noticed that women and people living in urban areas were more prone to sleep problems. Additionally, being informed about the infection of a close person was a factor influencing sleep disturbances [27]. In response to the findings of Voitsidis et al., similar studies were conducted in France by Kokou-Kpolou et al., which also showed the existence of insomnia problems. However, the results obtained were half as low as in the study by Voitsidis et al., but similar to the criteria observed in the Chinese and Italian populations [48]. It was also confirmed that the main predisposing factors for the occurrence of sleep disorders were concerns related to COVID-19 and loneliness.

The analysis of our own research using the AIS questionnaire indicated an exacerbation of insomnia symptoms during the second lockdown, both in women and men, and their reduction during the third lockdown. However, by comparison, a continuous upward trend in the examined parameter was observed. A higher level of sleep problems was characteristic of female respondents. Our data indicated an exacerbation of insomnia symptoms during the second lockdown, followed by a slight decrease during the third lockdown, with a continuous upward trend over the study period. Sleep problems were more pronounced in women. These findings suggest that sleep disturbances should be considered an important component of therapeutic strategies for supporting mental health in seniors during pandemics.

## 5. Conclusions

During the coronavirus pandemic, the level of physical activity among the surveyed group of seniors was above average and showed an upward trend. Due to the introduced restrictions primarily associated with limiting social contacts, seniors spent more time engaging in recreational activities. Unfortunately, the pandemic exacerbated symptoms of anxiety, aggression, and insomnia, particularly in the female respondent group. Meanwhile, symptoms of depression remained relatively stable among women, while they decreased in the male group. These observations were confirmed in the self-assessment of seniors’ health, where the psychological component of health deteriorated during the pandemic, while the physical component improved.

In general, no statistically significant correlations were observed between the level of physical activity and quality of life, anxiety, depression, aggression, or sleep disorders in any of the surveyed groups across the three studies. This indicated that, for the majority of participants, physical activity did not demonstrate a measurable association with psychological or functional outcomes. These isolated significant results highlight the potential moderating role of sex and study context in the relationship between physical activity and depression, warranting further investigation in larger, more diverse cohorts.

During the COVID-19 pandemic, seniors maintained an above-average level of physical activity, with increased engagement in recreational activities despite social restrictions. Anxiety, irritability, and insomnia worsened, particularly among women, while depression remained stable in women and decreased in men, reflecting changes in the psychological component of self-assessed health. No consistent associations were found between physical activity and quality of life or mental health outcomes, although isolated correlations suggest potential sex- and context-specific effects.

Limitations of the study: This study is limited by its observational design, absence of a control group, convenience sampling, lack of power analysis, heterogeneity of the age group, and limited control of confounding variables. Despite statistical correction, the risk of type I error cannot be fully excluded.

## Figures and Tables

**Table 1:** The characteristics of the study groups—Study I, II, and III.

Variable	Sex	Study I				Study II				Study III			
		X (SD)	Me	Q1	Q3	X (SD)	Me	Q1	Q3	X (SD)	Me	Q1	Q3
Age [years]	W *n* = 32	69.59 (7.97)	69	62.5	74	69.59 (7.97)	69	62.5	74	70.36 (7.46)	69	63	77
	M *n* = 21	69.67 (7.88)	67	65	73	69.67 (7.88)	67	65	73	70.19 (7.97)	68	65	75
Height [m]	W *n* = 32	1.64 (0.07)	1.64	1.59	1.68	1.64 (0.07)	1.64	1.59	1.68	1.64 (0.07)	1.64	1.59	1.68
	M *n* = 21	1.73 (0.05)	1.72	1.7	1.74	1.73 (0.05)	1.72	1.7	1.74	1.73 (0.05)	1.72	1.7	1.74
Weight [kg]	W *n* = 32	71.78 (13.26)	69	61.5	80	71.78 (13.26)	69	61.5	80	74.09 (13.67)	70	65	82.5
	M *n* = 21	87.29 (13.27)	87	76	97	87.29 (13.27)	87	76	97	87.57 (13.82)	87	76	98
BMI	W *n* = 32	31.89 (4.79)	31.79	27.9	34.91	31.89 (4.79)	31.79	27.9	34.91	32.54 (4.74)	32.5	27.98	35.1
	M *n* = 21	33.57 (4.3)	32.56	30.44	36.62	33.57 (4.3)	32.56	30.44	36.62	33.65 (4.47)	33.22	30.44	37

*n*—size of the study group, W—women, M—men, X—mean, SD—standard deviation, Me—median, Q1—lower quartile, Q3—upper quartile, BMI—body mass index.

**Table 2:** The Modified Baecke Questionnaire for Older Adults—Studies I, II, and III.

Variable	Sex	Study I			Study II			Study III			*p*
		Me Q1 Q3	X (Min–Max)	SD	Me Q1 Q3	X (Min–Max)	SD	Me Q1 Q3	X (Min–Max)	SD	
General activities score (HPA)	W *n* = 32	3.39 2.61 5.36	5.23 (1.6–22.51)	5.06	2.73 2.05 4.89	4.75 (1.1–26.88)	5.59	3.96 2.22 7.24	5.34 (1.8–23.87)	4.65	0.07
	M *n* = 21	4.69 2.92 12.12	7.39 (1.1–16.78)	5.59	4.19 2 11.52	6.68 (0.6–16.78)	5.54	7.02 2.92 10.41	7.83 (0.6–24.57)	6.48	0.09

*n*—size of the study group, W—women, M—men, X—mean, SD—standard deviation, Me—median, Q1—lower quartile, Q2—median, Q3—upper quartile, min—minimum value, max—maximum value.

**Table 3:** Short-Form 36 Health Status Questionnaire (SF 12v2)—Studies I, II, and III.

Variable	Sex	Study I			Study II			Study III			*p*
		Me	X (Min–Max)	SD	Me	X (Min–Max)	SD	Me	X (Min–Max)	SD	
MCS	W *n* = 32	79.17	76.2 (15–100)	17.29	70.83	70.66 (15–93.33)	17.13	75	72.92 (40–100)	15.79	0.02 *
	M *n* = 21	81.67	80.79 (25–100)	18.29	81.67	79.29 (25–100)	19.83	83.33	79.44 (38.3–100)	19.89	0.64
PCS	W *n* = 32	65	62.97 (4.17–95.83)	19.27	63.75	63.07 (4.17–95.83)	19.52	62.5	61.3 (6.67–95.83)	20.5	0.89
	M *n* = 21	66.67	66.94 (23.3–95.83)	19.08	66.67	66.78 (23.3–95.83)	19.87	68.33	67.69 (23.3–95.83)	19.81	0.73

*n*—size of the study group, W—women, M—men, Me—median, X—mean, min—minimum value, max—maximum value, SD—standard deviation, * *p* < 0.05, MCS—mental health component, PCS—physical health component.

**Table 4:** Hospital Anxiety and Depression Scale (HADS-m) and Athens Insomnia Scale (AIS) comparison of psychological parameters between women and men across subsequent studies.

Variable	Sex	Study I			Study II			Study III			*p*
		Me	X (Min–Max)	SD	Me	X (Min–Max)	SD	Me	X (Min–Max)	SD	
Anxiety	W *n* = 32	7	7.5 (2–15)	2.76	9	8.78 (4–17)	2.78	8.5	8.44 (4–15)	2.46	0.004 *
	M *n* = 21	7	6.57 (3–10)	1.89	7	7 (3–10)	1.54	2	6.95 (0–5)	1.32	0.66
*p*			0.03 *			0.002 *			0.03 *		
Depression	W *n* = 32	12	11.8 (7–15)	1.59	12	11.3 (6–15)	1.86	12	11.44 (6–14)	1.76	0.64
	M *n* = 21	12	11.19 (7–13)	1.47	11	10.76 (7–13)	1.84	10	10.24 (5–15)	2.3	0.02 *
*p*			0.18			0.79			0.94		
Aggression	W *n* = 32	2	2.03 (0–4)	1.4	3	2.56 (0–5)	1.46	2.5	2.47 (0–5)	1.68	0.03 *
	M *n* = 21	1	1.57 (0–5)	1.5	2	1.95 (0–5)	1.75	2	1.57 (0–5)	1.33	0.41
*p*			0.18			0.07			0.46		
Insomia	W *n* = 32	6	5.47 (0–11)	3.1	6.5	6.34 (0–16)	3.87	5.5	5.7 (0–12)	3.09	0.17
	M *n* = 21	3	3.48 (0–13)	3.04	3	4.33 (0–15)	3.77	3	3.62 (0–15)	3.49	0.14
*p*			0.06			0.41			0.03 *		

*n*—size of the study group, W—women, M—men, Me—median, X—mean, min—minimum value, max—maximum value, SD—standard deviation, * *p* < 0.05.

**Table 5:** Spearman’s rank correlations between the level of physical activity and quality of life, anxiety, depression, aggression, and insomnia across three study waves.

Variable	Sex	Study I *R*	Study II *R*	Study III *R*
MCS	W *n* = 32	0.03	0.017	−0.102
	M *n* = 21	0.27	0.217	0.397
PCS	W *n* = 32	−0.081	0.11	−0.077
	M *n* = 21	0.124	0.137	0.379
Anxiety	W *n* = 32	0.002	0.082	−0.047
	M *n* = 21	0.202	0.098	0.082
Depression	W *n* = 32	0.364 *	0.331	0.225
	M *n* = 21	0.201	0.398	0.487 *
Aggression	W *n* = 32	−0.087	−0.091	0.091
	M *n* = 21	0.127	−0.043	−0.068
Insomia	W *n* = 32	0.128	0.31	0.118
	M *n* = 21	−0.077	−0.25	−0.113

*n*—sample size; W—women; M—men; R—Spearman correlation coefficient. Statistical significance was set at * *p* < 0.05. No correction for multiple comparisons was applied.

## Data Availability

The raw data supporting the conclusions of this article will be made available by the authors on request.
